# Anti-Photoaging Effects of a Polysaccharide from *Kappaphycus alvarezii* In Vitro and In Vivo

**DOI:** 10.3390/md24020087

**Published:** 2026-02-21

**Authors:** Yixuan Lai, Yuan Wang, Abdul Mueed, Peng Shu, Lijun You, Jiangming Zhong

**Affiliations:** 1 School of Food Science and Engineering, South China University of Technology, Guangzhou 510640, China; yxlaiiii@163.com (Y.L.); amueed3723@yahoo.com (A.M.); 2 HBN Research Institute and Biological Laboratory, Shenzhen Hujia Technology Co., Ltd., Shenzhen 518038, China; wangyuan@hbn.cn (Y.W.); zhongjiangming@hbn.cn (J.Z.); 3 Guangdong Engineering Technology Research Center for Functional Skincare Innovation, Shenzhen Hujia Technology Co., Ltd., Shenzhen 518052, China

**Keywords:** anti-photoaging, *Kappaphycus alvarezii*, polysaccharides, UV, effect

## Abstract

The red alga *Kappaphycus alvarezii* is a rich source of polysaccharides, but their high molecular weight limits skin permeability and bioavailability. To address this, we employed a free-radical degradation method to produce a low-molecular-weight polysaccharide, KP-90. Evaluation in UVB-irradiated HaCaT cells and UVA-irradiated human dermal fibroblasts demonstrated that KP-90 significantly enhanced cell viability and mitigated oxidative stress by suppressing reactive oxygen species and malondialdehyde, while restoring antioxidant enzymes (SOD, CAT and GSH-Px). Furthermore, KP-90 downregulated matrix metalloproteinases (MMP-1, -3, -9) and pro-inflammatory cytokines (TNF-α, IL-6, IL-1β), thereby reducing extracellular matrix degradation and inflammation. These in vitro findings were corroborated in a UVB/UVA-irradiated nude mice model, where KP-90 alleviated epidermal hyperplasia, increased collagen I and hyaluronic acid synthesis, and improved visible signs such as wrinkles and skin laxity. These findings identify KP-90 against skin photoaging and provide a strategic approach for valorization underexploited marine biomass.

## 1. Introduction

Photoaging is a form of extrinsic skin aging caused by repeated exposure to natural or artificial ultraviolet (UV) radiation [1]. Clinically, it is characterized by dryness, roughness, deepening wrinkles, skin laxity, uneven pigmentation, and telangiectasia. In severe cases, it may even lead to skin cancer and related pathologies [2]. The main mechanisms underlying photoaging include oxidative stress, DNA damage and mutation, degradation of the extracellular matrix (ECM) by matrix metalloproteinases (MMPs), inflammatory responses, melanogenesis, and gut microbiota dysbiosis. At the cellular level, photoaging manifests as cellular senescence, reduced cell viability, and alterations in ECM composition [3]. Targeting ECM metabolism through therapeutic interventions, such as repairing ECM structural damage and enhancing skin barrier function, has become a key strategy for mitigating and preventing photoaging damage. For instance, dieckol isolated from *Eisenia bicyclis* extract was applied to nude mice repeatedly exposed to medium-wave ultraviolet B (UVB). It was found to exert anti-photoaging effects by modulating ECM components, including suppressing UVB-induced collagen degradation and the expression of MMP-1, -3, and -9 [4]. Similarly, DSFP-45, a polysaccharide derived from *Sargassum fusiforme*, reduced photoaging damage by regulating ECM metabolism. Studies showed that it significantly increased the content and expression of collagen I while decreasing levels of pro-inflammatory cytokines such as interleukin-1 beta, interleukin-6, and tumor necrosis factor-α, suggesting potential anti-photoaging activity in skin cells. In addition, *Blumea balsamifera* oil effectively protected fibroblasts from UV-induced photoaging by alleviating inflammation, enhancing the synthesis of antioxidant enzymes, and slowing the degradation of ECM proteins [5]. Numerous studies have confirmed that various active ingredients can alleviate photoaging by modulating ECM metabolism and strengthening skin barrier function.

Preventing and reducing photoaging damage remains a major focus in the cosmetics industry. In terms of raw materials, many brands are turning to marine resources, utilizing biotechnology to obtain safe and effective active ingredients from seaweeds. *Kappaphycus alvarezii* (KP) is a red algae species belonging to the family *Solieriaceae*, widely cultivated in Asian countries such as Indonesia, the Philippines, China, Vietnam, and Malaysia [6]. It is a high-carbohydrate (averaging 51%), low-protein, and low-fat seaweed, well-known for its rich κ-carrageenan content and recognized as an economically important tropical commercial seaweed [7]. In recent years, its bioactivities have attracted increasing research interest. Wu et al. extracted and purified sulfated polysaccharides from *Kappaphycus alvarezii* (KSP) via ultrasound-assisted hot water extraction. KSP treatment in RBL-2H3 cells significantly inhibited degranulation and histamine release, demonstrating notable anti-allergic activity in vitro [8]. Moreover, *Kappaphycus alvarezii* extract was found to exert anti-inflammatory effects in asthma-induced rats by downregulating TNF-α, IL-4, and nuclear factor-κB. Other bioactivities, such as antioxidant, antimicrobial, anticancer, and cardiovascular protective effects, have also been reported [9]. Given these promising properties, this study aims to explore the potential anti-photoaging activity of KP from the perspective of ECM metabolism regulation.

It has been reported that the high molecular weight and viscosity of polysaccharides often hinder their bioavailability, thereby limiting their further development [10]. Previous studies have shown that polysaccharides extracted from *Kappaphycus alvarezii* via traditional hot-water extraction can have a molecular weight of up to 260 kDa. These polysaccharides exhibit high viscosity and tend to form solid lumps at room temperature, which restricts their practical application. Degraded polysaccharides, owing to their reduced molecular size, generally demonstrate enhanced bioactivity. Research indicates that lower molecular weight correlates with higher skin permeation efficiency [11]. Current approaches for polysaccharide depolymerization include physical methods, chemical methods, enzymatic methods, and free radical-based degradation. Among these, free radical degradation has garnered increasing attention due to its high efficiency, cost-effectiveness, and environmental friendliness. In polysaccharide degradation systems, hydroxyl radicals are the most widely used, exhibiting strong depolymerizing activity [12]. The hydroxyl radicals generated through these methods can significantly reduce the molecular weight of polysaccharides and improve their bioactivity. In our preliminary research, we found that the UV/H_2_O_2_ system showed excellent efficacy in degrading polysaccharides from *Sargassum fusiforme* and enhancing their biological activity [13], which provided a valuable foundation for the present study.

Therefore, in this study, we applied UV/H_2_O_2_ technology to prepare a low-molecular-weight polysaccharide from *Kappaphycus alvarezii* and investigated its anti-photoaging effects through in vitro models using epidermal and dermal cells, as well as an in vivo mice model. We systematically elucidated its mechanism of action via regulation of ECM metabolism. This work represents the first application of UV/H_2_O_2_ technology in the preparation of KP polysaccharides, offering a green, efficient, and sustainable approach to enhance the utilization of algal biomass. Furthermore, it is the first study to explore the anti-photoaging activity of KP extracts, aiming to unlock its potential in skincare and promote the comprehensive development and utilization of marine bio-resources, thereby contributing to the growth of the blue bioeconomy.

## 2. Results

### 2.1. In Vitro Skin Permeation of KP-90

**Figure 1:**
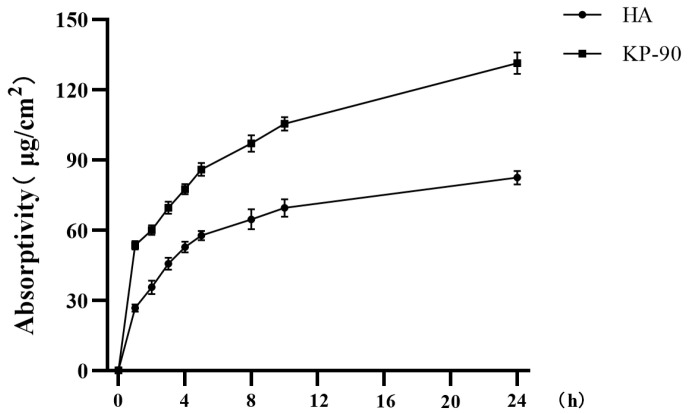
Transdermal absorption rates of KP-90 and hyaluronic acid (HA) in vitro.

The skin permeation behavior of KP-90 was evaluated using a Franz diffusion cell model. Neonatal porcine skin, which closely resembles human skin in structure, was selected as the membrane. The concentration of 10 mg/mL was selected based on preliminary cytotoxicity assays, which confirmed that both KP-90 and HA at this concentration were non-cytotoxic and fully soluble, allowing for a valid comparative assessment of skin permeation. Figure 1 compares the permeation profiles of KP-90 and hyaluronic acid (Mw 3–5 kDa) at the same concentration. KP-90 exhibited superior skin permeation within the first hour, and after 24 h, its cumulative absorption was nearly twice that of hyaluronic acid. These vitro results demonstrate the superior skin permeation capacity of KP-90 through neonatal porcine skin under the tested conditions, which validates the rationale for its topical application prior to proceeding with in vitro and vivo efficacy evaluations.

### 2.2. KP-90 Attenuates UVB-Induced Damage in HaCaT Cells

**Figure 2:**
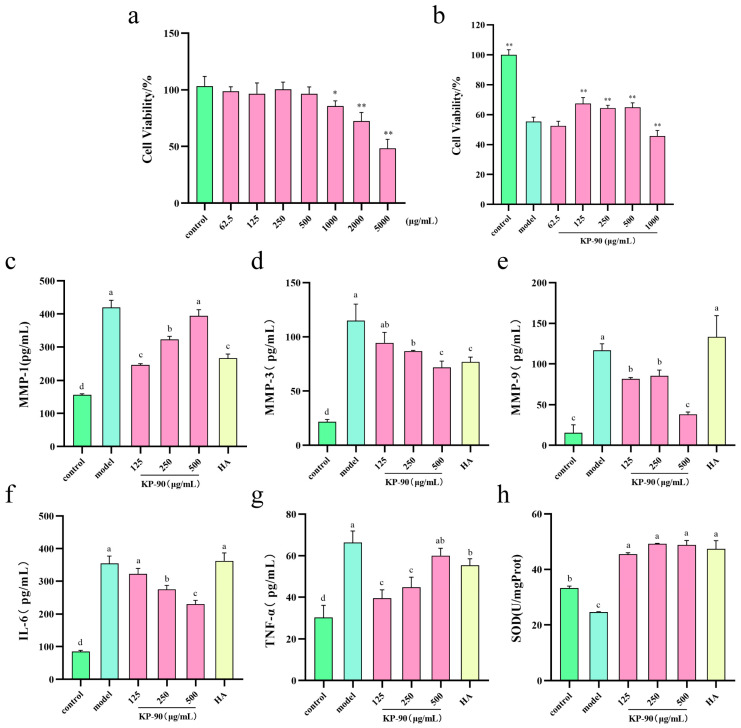
The cell viability, secretion level and enzyme activity of UVB-induced HaCaT cells after KP-90 treatment. (**a**) Cell viability, (**b**) Cell viability (irradiation), (**c**) MMP-1 level, (**d**) MMP-3 level, (**e**) MMP-9 level, (**f**) IL-6 level, (**g**) TNF-α level, (**h**) SOD activity. * *p* < 0.05, ** *p* < 0.01, versus the model group. Different letters showed significant differences (*p* < 0.05) between each group.

We first assessed the safety and efficacy of KP-90 in skin cells. Cytotoxicity test indicated that KP-90 at concentrations exceeding 1000 μg/mL significantly reduced cell viability compared to the control group (Figure 2a). Following UVB irradiation, the cell survival rate in the model group decreased to 55% relative to the control (Figure 2b), indicating successful induction of photoaging damage in HaCaT cells. Treatment with different concentrations of KP-90 showed varying protective effects. Concentrations of 125, 250, and 500 μg/mL alleviated UVB-induced damage, whereas at 1000 μg/mL, cell viability was significantly lower than that in the model group, suggesting cytotoxic effects at this higher concentration. Therefore, the concentrations of 125, 250, and 500 μg/mL were selected for subsequent cellular assays.

### 2.3. Effects of KP-90 on MMP-1, MMP-3, and MMP-9 Expression in HaCaT Cells

Overactivation of MMPs is a hallmark of photoaging, as they degrade structural proteins in the dermis, leading to wrinkles and skin laxity [14]. UVB irradiation significantly upregulated the production of MMP-1, MMP-3, and MMP-9 in HaCaT cells (Figure 2c–e). KP-90 at 125 and 250 μg/mL markedly suppressed MMP-1 synthesis, thereby reducing extracellular matrix degradation and exhibiting anti-photoaging activity (Figure 2c). The inhibition of MMP-3 exhibited a concentration-dependent response, with 500 μg/mL KP-90 significantly reducing its secretion, comparable to the effect of 500 μg/mL hyaluronic acid (Figure 2d). Hyaluronic acid was selected as the positive control. As a polysaccharide with well-established bioactivity, hyaluronic acid is widely employed in efficacy comparison studies involving polysaccharides [15]. Moreover, 500 μg/mL KP-90 significantly downregulated MMP-9 expression, mitigating cellular damage and demonstrating notable anti-photoaging properties (Figure 2e).

### 2.4. Effects of KP-90 on IL-6 and TNF-α Expression in HaCaT Cells

UV radiation induces ROS that stimulate the release of pro-inflammatory cytokines, triggering skin inflammation and impairing epidermal barrier function. Pro-inflammatory factors such as TNF-α and IL-6 can downregulate the expression of filaggrin and loricrin in keratinocytes, resulting in thinning of the stratum corneum and manifestations such as dryness, roughness, and sensitivity [16]. UVB irradiation elevated the levels of IL-6 and TNF-α in HaCaT cells, confirming inflammatory photoaging (Figure 2f,g). Treatment with 500 μg/mL KP-90 significantly reduced IL-6 secretion, outperforming the same concentration of hyaluronic acid (Figure 2f). Additionally, KP-90 effectively suppressed TNF-α release in a concentration-dependent manner, thereby alleviating photoaging-associated inflammatory injury (Figure 2g).

### 2.5. Effect of KP-90 on SOD Activity in HaCaT Cells

SOD plays a crucial role in neutralizing superoxide radicals (O_2_^−^), protecting biomacromolecules from oxidative damage and maintaining cellular redox homeostasis [17]. As shown in Figure 2h, SOD activity was significantly lower in the model group than in the control, indicating disruption of the oxidative–antioxidative balance following UVB exposure. KP-90 treatment restored SOD activity across all tested concentrations, with effects comparable to those of 500 μg/mL hyaluronic acid.

### 2.6. KP-90 Protects HDF Cells from UVA-Induced Damage

**Figure 3:**
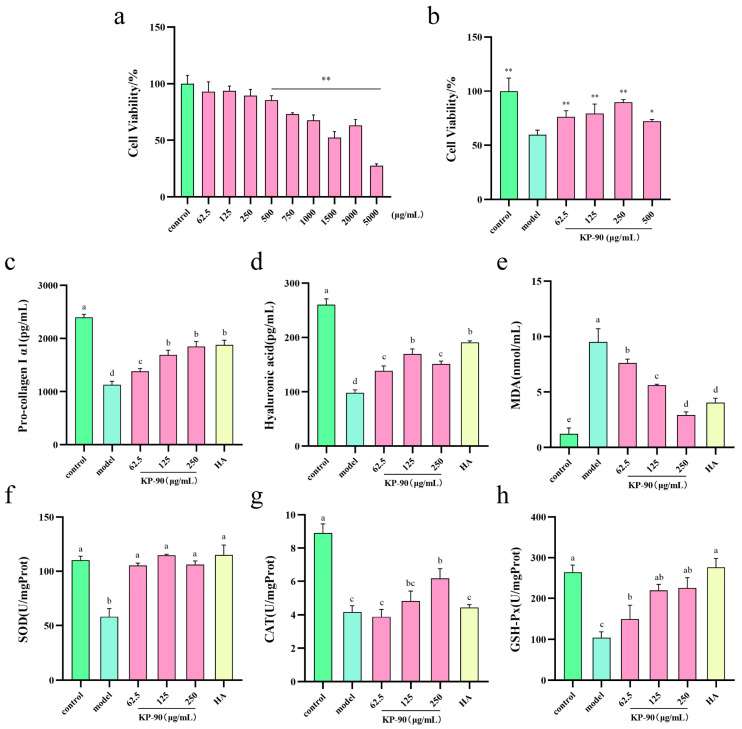
The cell viability, secretion level and enzyme activity of UVA-induced HDF cells after KP-90 treatment. (**a**) Cell viability, (**b**) Cell viability (irradiation), (**c**) pro-collagen I α1 level, (**d**) hyaluronic acid level, (**e**) MDA level, (**f**) SOD activity, (**g**) CAT activity, (**h**) GSH-Px activity. * *p* < 0.05, ** *p* < 0.01, versus the model group. Different letters showed significant differences (*p* < 0.05) between each group.

In HDF cells, KP-90 demonstrated cytotoxicity at concentrations above 500 μg/mL compared to the control group (Figure 3a). UVA irradiation reduced cell viability to 60% in the model group compared to the control group, confirming successful photoaging induction (Figure 3b). Treatment with various concentrations of KP-90 significantly alleviated irradiation-induced damage and improved cell survival, although the protective effect diminished at 500 μg/mL. Based on these results, concentrations of 62.5, 125, and 250 μg/mL were selected for subsequent experiments.

### 2.7. Effects of KP-90 on Collagen and Hyaluronic Acid in HDF Cells

Collagen provides structural support, strength, and elasticity to human skin. Type I collagen, the most abundant form, is primarily synthesized and secreted by dermal fibroblasts. It is derived from the cleavage of procollagen type I, a heterotrimeric molecule consisting of two α1 chains and one α2 chain, which is secreted into the extracellular matrix [18]. UVA irradiation significantly reduced the secretion of procollagen type I α1 chain secreted by HDF cells. In contrast, KP-90 treatment markedly increased its secretion in a concentration-dependent manner, with 250 μg/mL KP-90 showing efficacy comparable to that of 250 μg/mL hyaluronic acid (Figure 3c).

Hyaluronic acid levels reflect skin health, youthfulness, and functional integrity. UV radiation promotes hyaluronic acid degradation and inhibits its synthesis by fibroblasts, leading to a sharp decline in content and impaired skin barrier function [19]. Treatment with KP-90 increased hyaluronic acid levels in all experimental groups, indicating its anti-photoaging potential via enhancement of hyaluronic acid production (Figure 3d).

### 2.8. Effects of KP-90 on Antioxidant Indicators in HDF Cells

UV irradiation induces the formation of oxygen free radicals in human skin, which attack polyunsaturated fatty acids in biological membranes and generate lipid peroxides such as malondialdehyde (MDA). MDA levels reflect the extent of free radical-induced cellular damage. UVA irradiation significantly increased MDA content in HDF cells, indicating severe oxidative injury. KP-90 treatment significantly reduced MDA levels in a concentration-dependent manner (Figure 3e).

The enzymatic antioxidant system, comprising SOD, CAT, and GSH-Px, plays a major role in scavenging oxygen free radicals and maintaining redox homeostasis. SOD catalyzes the conversion of excess O_2_^−^ into H_2_O_2_, which is then decomposed into harmless products by CAT and GSH-Px, thereby blocking free radical chain reactions and protecting extracellular matrix components such as collagen and elastic fibers [20]. UVA irradiation significantly decreased the activities of SOD, CAT, and GSH-Px in HDF cells. KP-90 treatment significantly restored the activity of all three enzymes in a concentration-dependent manner, thereby mitigating ROS-induced ECM degradation (Figure 3f–h).

### 2.9. Effect of KP-90 on Intracellular ROS Levels in HDF Cells

**Figure 4:**
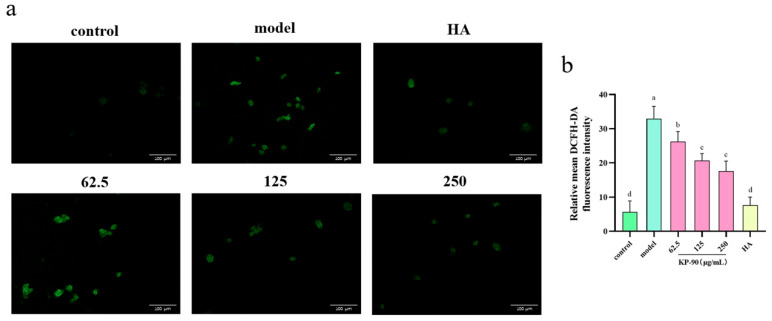
ROS fluorescence staining of UVA-induced HDF cells after KP-90 treatment. (**a**) Staining images. (**b**) ROS fluorescence intensity. Different letter showed significant differences (*p* < 0.05) between each group.

As shown in Figure 4a, UVA irradiation significantly increased intracellular ROS levels in HDF cells. Elevated ROS not only attack cellular components but also directly oxidize and degrade dermal structural proteins such as collagen and elastin. Treatment with KP-90 effectively suppressed ROS generation in a dose-dependent manner (Figure 4b).

### 2.10. Apparent Morphology and Skin Hydration in Photoaged Mice

**Figure 5:**
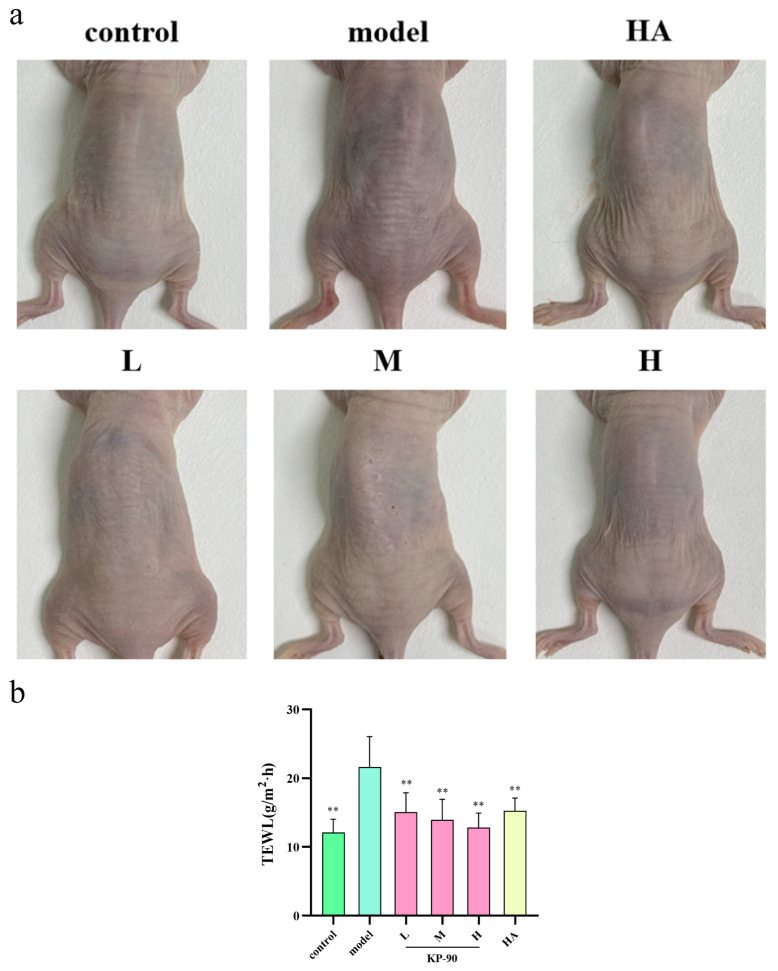
Skin absorption and water retention effect of KP-90 treatment. (**a**) Apparent morphology of mice skin. (**b**) TEWL of skin in photoaging mice. ** *p* < 0.01, versus the model group.

Chronic ultraviolet exposure impairs the skin barrier, leading to dryness, loss of elasticity, and sagging [21]. As shown in Figure 5a, skin in the control group remained elastic, smooth, and firm, whereas the model group exhibited significant roughness and wrinkling, confirming successful induction of photoaging. Topical application of KP-90 alleviated these symptoms in a dose-dependent manner; the high-dose group showed visibly diminished wrinkling and sagging compared to the model group. Skin in the HA group was relatively smooth and significantly improved over the model group, though still looser than that of the control group. Transepidermal Water Loss (TEWL) reflects the rate of water evaporation from the skin and is used to evaluate skin barrier integrity. UV irradiation significantly increased TEWL in the model group relative to the control. KP-90 treatment significantly reduced TEWL values compared to the model group, suggesting that it mitigates UV-induced barrier damage and promotes barrier recovery (Figure 5b).

### 2.11. Skin Staining Results in Mice

**Figure 6:**
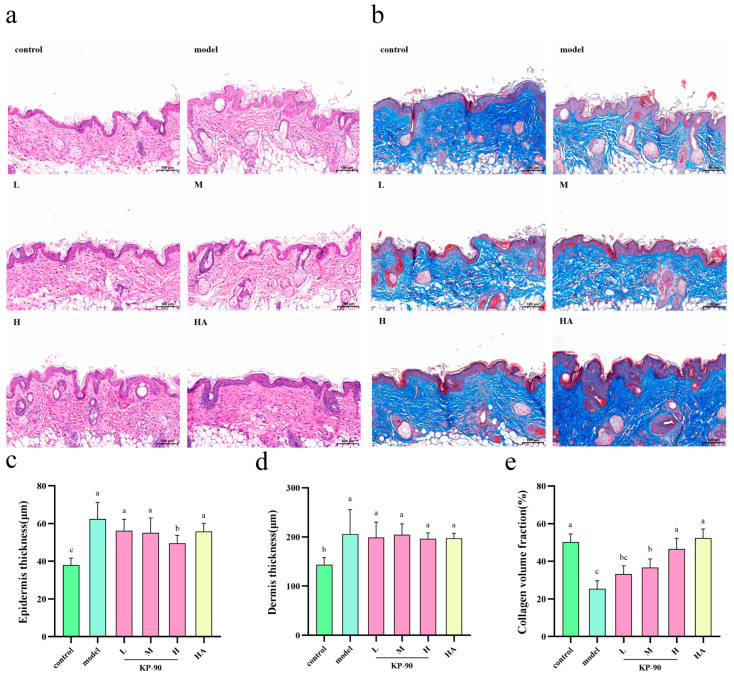
Effect of KP-90 on UV-induced photoaged mice skin by H&E and Masson staining. (**a**) HE staining, (**b**) Masson staining, (**c**) Epidermis thickness, (**d**) Dermis thickness, (**e**) Collagen volume fraction. Different letters showed significant differences (*p* < 0.05) between each group.

The effect of irradiation on epidermal and dermal thickness was investigated using hematoxylin and eosin (H&E) staining. As shown in Figure 6a, skin sections from the control group exhibited a clear boundary between the epidermis and dermis, with collagen fibers in the dermis arranged in a compact and orderly manner. Following UVA/UVB irradiation, the model group showed a significant increase in epidermal thickness, disorganized and loosely packed dermal collagen fibers, a blurred dermo-epidermal junction, and disrupted structures such as sebaceous glands. Treatment with KP-90, however, led to considerable improvement in these parameters. Epidermal hyperplasia was suppressed, disrupted collagen fibers were repaired, and the dermo-epidermal junction appeared clearer compared to the model group, with more pronounced effects observed at higher KP-90 concentrations. Additionally, topical application of high-dose hyaluronic acid also ameliorated the UV-induced disorganization of collagen fibers.

Epidermal and dermal thickness were measured from H&E-stained sections using ImageJ 1.x software, and the results are presented in Figure 6c,d. Compared to the control group, the model group exhibited a significant increase in epidermal thickness (Figure 6c). While treatment with KP-90 and hyaluronic acid tended to reduce epidermal thickness, the differences were not statistically significant, except in the H group. Regarding dermal thickness (Figure 6d), an increase was observed in the model group compared to the control. However, no significant differences in dermal thickness were found between the KP-90-treated groups and the model group.

The impact of irradiation on collagen fibers was further assessed by Masson’s trichrome staining. As demonstrated in Figure 6b, skin sections from the control group displayed intense blue staining, indicating a high collagen content in the dermis. The collagen exhibited a wavy, well-organized, dense, and concentrated distribution. In contrast, the model group showed lighter and less dense blue staining, consistent with the H&E findings, along with a fragmented collagen network. Treatment with KP-90 markedly improved these morphological changes. With increasing concentrations of KP-90, the collagen fibers became progressively more compact and orderly, transforming from a disrupted and loose state in the model group to a denser and more organized arrangement.

The collagen volume fraction (CVF), determined by semi-quantitative analysis using ImageJ 1.x, is shown in Figure 6e. A significant decrease in CVF was observed in the model group compared to the control group. In contrast, the M and H KP-90 treatment groups showed a significant increase in CVF compared to the model group, indicating that KP-90 effectively mitigated the adverse effects of UV irradiation on collagen. Furthermore, topical application of high-dose hyaluronic acid significantly promoted collagen fiber synthesis, demonstrating that high-dose hyaluronic acid also exerts protective effects against UV-induced collagen degradation, which is consistent with its well-established role in skin hydration and repair.

### 2.12. Secretion of TNF-α, IL-1β, and IL-6

**Figure 7:**
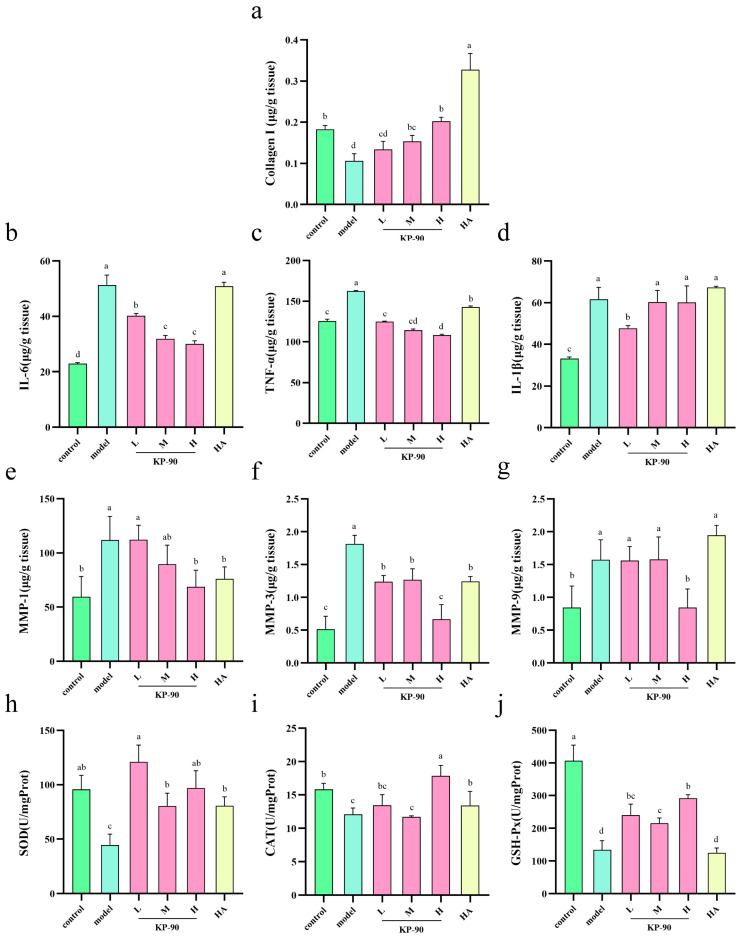
The secretion level of UV-induced mice after KP-90 treatment. (**a**) Collagen I content, (**b**) IL-6 level, (**c**) TNF-α level, (**d**) IL-1β level, (**e**) MMP-1 level, (**f**) MMP-3 level, (**g**) MMP-9 level, (**h**) SOD activity, (**i**) CAT activity, (**j**) GSH-Px activity. Different letters showed significant differences (*p* < 0.05) between each group.

As shown in Figure 7b–d, UV irradiation induced a marked elevation in the levels of TNF-α, IL-6, and IL-1β in the skin of the model group, indicating inflammatory damage. KP-90 treatment groups exhibited varying degrees of reduction in TNF-α and IL-6 content, suggesting that KP-90 alleviate UV-induced skin injury. The suppressive effect of KP-90 on the expression of these cytokines was more pronounced than that of hyaluronic acid and demonstrated a dose-dependent trend (Figure 7b,c). As shown in Figure 7d, only the low-dose KP-90 group exhibited a statistically significant reduction in IL-1β levels compared to the model group. No significant differences were observed in the medium-dose, high-dose, or HA groups.

### 2.13. Collagen I Content and Secretion of MMP-1, MMP-3, and MMP-9 in Mice Skin

UV irradiation led to a reduction in type I collagen content in the skin of the model group. Consistent with the in vitro findings, KP-90 treatment in vivo significantly increased type I collagen content in the skin, particularly at medium and high doses, indicating that topical application of KP-90 at higher doses promotes collagen synthesis and helps counteract the loss of extracellular matrix components (Figure 7a).

As shown in Figure 7e–g, UV irradiation significantly upregulated the expression of MMP-1, MMP-3, and MMP-9 in the model group relative to the control, accelerating ECM degradation and contributing to skin laxity and wrinkle formation. The high-dose KP-90 significantly reduced the expression of both MMP-1 and MMP-9 compared to the model group, demonstrating that KP-90 effectively inhibits the synthesis and secretion of these proteases, thereby reducing collagen breakdown (Figure 7e,g). All treatment groups showed a decreasing trend in MMP-3 levels relative to the model group, suggesting a general suppressive effect on MMP-3 expression. Among them, the high-dose KP-90 group exhibited the most pronounced inhibition, indicating superior efficacy in suppressing MMP-3 synthesis and secretion compared to the same dose of hyaluronic acid (Figure 7f).

### 2.14. Activities of SOD, CAT, and GSH-Px

UV irradiation damaged skin cells and reduced the activities of SOD, CAT, and GSH-Px. KP-90 treatment groups showed varying degrees of increase in SOD activity (Figure 7h). CAT activity was significantly elevated in the high-dose KP-90 group compared to the model group, and this effect was superior to that of the HA group. No significant improvement in CAT activity was observed in the other treatment groups (Figure 7i). In contrast, all three doses of KP-90 significantly increased GSH-Px activity relative to the model group (Figure 7j).

## 3. Discussion

UV radiation, particularly UVA and UVB, is the primary environmental factor responsible for skin photoaging. UVA accounts for over 90% of the UV radiation that reaches the Earth’s surface. It possesses strong penetrating ability, reaching the dermis where it can induce cellular damage and collagen degradation, leading to a range of photoaging-related issues [22]. In contrast, UVB, though more energetic, constitutes less than 5% of terrestrial UV radiation and has weaker skin penetration, primarily affecting the epidermal layer. Nevertheless, even limited exposure to UVB can cause significant damage to epidermal cells such as keratinocytes, resulting in photoaging symptoms [22]. Numerous studies have demonstrated that certain polysaccharides can mitigate UV-induced skin damage. For example, oral administration of Galacto-Oligosaccharides was shown to reduce UVB-induced wrinkle formation in hairless mice by modulating extracellular matrix metabolism [21]. Topical application of Chitosan Oligosaccharides alleviated collagen fiber damage, increased the relative and total content of type I collagen, and significantly improved both macroscopic and histopathological skin damage in mice [23].

Polysaccharides from *Kappaphycus alvarezii* have also been reported to exhibit various bioactivities, including anti-inflammatory, antioxidant, antimicrobial, and anticancer effects in different models [9]. Owing to its excellent bioactivity, this study aimed to investigate whether it could exert reparative effects in a UV-induced photoaging damage model from the perspective of modulating ECM-related component metabolism. The polysaccharide KP-90 was determined by gel permeation chromatography to have a relative molecular weight of 4 kDa. Compared with polysaccharides KP-0 (12 kDa) directly extracted by hot water from *Kappaphycus alvarezii*, KP-90 exhibits improved flow properties and has been shown in prior studies to achieve higher in vitro skin absorption efficiency. The sulfate content of KP-90 was determined to be 22.44 ± 2.72% using the barium chloride-gelatin method, slightly higher than that of KP-0 (20.00 ± 2.97%). FTIR absorption bands confirming the polysaccharides backbone. A more detailed structural characterization will be reported in a forthcoming publication. These data provide sufficient evidence that KP-90 is a low-molecular-weight sulfated polysaccharide, and probably related to the observed bioactivity. The sulfate groups are known to facilitate interactions with growth factors and ECM components, while the lower Mw improves skin permeation and receptor binding accessibility [15].

The skin permeation behavior of KP-90 was preliminarily evaluated using an in vitro Franz diffusion cell system. Hyaluronic acid with a molecular weight of 3–5 kDa was selected as a comparator. This molecular weight range is comparable to that of KP-90 (4.2 kDa), allowing for a fair comparison of their skin permeation and bioactivity. HA is a naturally occurring glycosaminoglycan in the skin ECM and is widely used as a benchmark ingredient in anti-aging and moisturizing studies due to its well-characterized effects on hydration and MMP suppression [13]. As shown in Figure 1, KP-90 exhibited superior skin permeation compared to hyaluronic acid, a phenomenon that may involve passive diffusion transport [24]. While the Franz diffusion cell assay provides valuable insight into the passive diffusion behavior of KP-90, it is important to acknowledge its limitations. This ex vivo model does not account for active transport, metabolic processes, or the dynamic clearance mechanisms present in living organisms. Therefore, the permeation efficiency observed in this study represents an estimate. Then in cell models, we found KP-90 demonstrated notable preventive efficacy in mitigating such damage while UV leading to reduced cell viability (Figure 2b and Figure 3b).

UV irradiation generates of ROS, which can accumulate and inflict irreversible cellular damage. This oxidative stress impairs DNA and protein structures, accelerates extracellular matrix degradation, promotes cellular senescence and apoptosis, and disrupting skin barrier function [25]. Furthermore, ROS attack polyunsaturated fatty acids in cell membranes, generating lipid peroxides such as MDA and exacerbating cellular damage [20]. In our study, KP-90 treatment in UVA-irradiated HDF cells significantly enhanced the activities of SOD, CAT, and GSH-Px. This leads to a marked reduction in both ROS and MDA levels, alleviating oxidative stress injury, and subsequent collagen degradation (Figure 2f–h). This finding aligns with previous work by Tian et al. who reported that Artesunate treatment in UVB-irradiated HaCaT cells increased SOD activity, reduced intracellular ROS generation, and alleviated photoaging damage [26].

Key histological features of skin photoaging include collagen fiber loss and aberrant elastin deposition. Type I collagen, the most abundant isoform, is synthesized and secreted by dermal fibroblasts as procollagen type I, which undergoes proteolytic cleavage to generate mature collagen that provides structural integrity and elasticity to the skin [27]. Type I collagen is derived from the proteolytic cleavage of procollagen type I, a heterotrimeric molecule composed of two α1 chains and one α2 chain [18]. Hyaluronic acid, a critical glycosaminoglycan in the dermal extracellular matrix, exhibits high water-retention capacity and synergizes with collagen and elastic fibers to maintain skin structure and hydration [28]. Elevated hyaluronic acid levels support skin plumpness, barrier integrity, and youthful appearance. Ultraviolet irradiation induces excessive reactive oxygen species, which fragment hyaluronic acid polymers, thereby impairing skin hydration, inducing dryness, and potentially exacerbating inflammatory responses [29].

Within cells, UV irradiation induces substantial generation of ROS (Figure 4), which directly damages collagen and hyaluronic acid, triggering their degradation. Concurrently, it activates relevant signaling pathways and upregulates the expression of MMP-1, MMP-3, and MMP-9. The overactivation of MMPs is a hallmark of photoaging. These enzymes directly degrade structural proteins within the dermis, resulting in aged skin manifestations such as wrinkles and laxity [14]. KP-90 alleviates photoaging damage by increasing the content of type I collagen and hyaluronic acid (Figure 3c,d) and suppressing the secretion of MMP-1, MMP-3, and MMP-9 (Figure 2c–e). Similar findings were reported by Hu et al. [15], who observed that *Sargassum fusiforme* purified polysaccharide P1 could inhibit the UVB-induced upregulation of MMP-1, MMP-3, and MMP-9 in a HaCaT cell model, thereby reducing collagen loss. In another study, Ren et al. [30] treated UV-induced mouse embryonic fibroblasts with a complex of j-ca3000 + CP (combined collagen peptide with j-carrageenan oligosaccharide) and found that it increased type I collagen synthesis, decreased MMP-1 expression, and restored the cells to near-normal levels. Yang et al. [31] demonstrated that cycloastragenol effectively elevated hyaluronic acid levels, significantly reduced the production of MMP-1, MMP-9, MMP-13, and ROS, enhanced type I collagen synthesis, and improved cell viability in both UVB-damaged HDF and HaCaT cells.

UV radiation not only stimulates ROS production and upregulates MMP secretion but also induces inflammatory responses [32]. Pro-inflammatory factors such as TNF-α, IL-1β, and IL-6 can suppress the expression of filaggrin, loricrin, and involucrin in keratinocytes, leading to a thinner stratum corneum, structural disorganization, and increased TEWL [16]. Additionally, certain inflammatory cytokines inhibit the synthesis of type I and III collagen, elastin, and fibronectin in dermal fibroblasts by modulating the TGF-β/Smad signaling pathway [33]. This results in dermal thinning, structural collapse, reduced elasticity, and the formation of deep wrinkles and skin laxity [34]. KP-90 mitigates UVB-induced photoaging damage in HaCaT cells by inhibiting the expression of TNF-α and IL-6 (Figure 2f,g), with certain concentrations exhibiting superior efficacy to hyaluronic acid. Similarly, Li et al. [34] used ELISA to measure the protein levels of TNF-α, IL-1β, and IL-6 in a UVA-induced HaCaT cell model and found that hesperidin exerted anti-photoaging effects by downregulating these inflammatory factors, which was further validated by quantitative reverse transcriptase PCR results.

In the animal experiment, hairless nude mice were used as the model organism, and a combined UVA-UVB irradiation protocol was applied to more accurately simulate the photoaging damage caused by natural sunlight. This model produces a milder, chronic form of damage that better reflects the daily photoaging process in most individuals. Chronic ultraviolet exposure leads to impaired skin barrier function, manifesting as dryness, loss of elasticity, and sagging [32]. As shown in Figure 5a, after seven weeks of irradiation, apparent changes in dorsal skin morphology were observed, including increased roughness, wrinkle formation, sagging, and localized erythema with scabbing. Treatment with KP-90 alleviated these symptoms. Similarly, Suh et al. [21] reported that dietary supplementation with galacto-oligosaccharides significantly reduced wrinkle area and average wrinkle length in UVB-exposed hairless mice compared to the UVB-only control group.

TEWL refers to the rate at which water naturally evaporates from the deeper layers of the skin through the epidermis to the external environment, and serves as an indicator of skin barrier integrity. Ultraviolet radiation disrupts the structure of the stratum corneum and damages keratinocytes, impairing the barrier’s ability to retain moisture and leading to elevated TEWL values [35]. The significant reduction in TEWL observed in the KP-90 groups compared to the model group suggests that topical application of KP-90 helps alleviate photoaging damage and maintains skin barrier integrity. In a related study, Kage et al. applied hyaluronic acid tetrasaccharide to hairless mice and found that it effectively promoted the recovery of skin function after UVA exposure, notably reducing the increased TEWL and attenuating epidermal hyperplasia [4].

UV irradiation induces epidermal hyperplasia, dermo-epidermal junction flattening, fibroblast proliferation, and inflammatory infiltration, with epidermal thickening serving as an adaptive response to limit further UV penetration [36]. KP-90 treatment attenuated epidermal hyperplasia (Figure 6c). However, its effect on dermal thickness was inconclusive. Although the model group exhibited increased dermal thickness with considerable heterogeneity—ranging from focal dermal atrophy with collagen degradation to loosened matrix with enlarged interstices—no statistically significant differences were observed between KP-90-treated and model groups. The reduced variability in treated groups suggests a potential protective effect against dermal tissue loss, though further investigation is warranted.

Masson’s trichrome staining demonstrated that UV irradiation markedly reduced dermal collagen content, likely via MMP-mediated degradation, contributing to skin laxity, wrinkle formation, and loss of elasticity. Concurrent epidermal thickening reflected keratinocyte hyperproliferation and delayed desquamation [37]. KP-90 treatment significantly increased collagen volume fraction in the M and H groups (Figure 6e), indicating effective mitigation of UV-induced collagen damage. These results are consistent with Her et al. [38], who reported that Oenanthe javanica extract attenuated epidermal hyperplasia and preserved dermal collagen architecture in UVB-irradiated mice.

Moving beyond in vitro cell studies, we further investigated changes in relevant factors in the in vivo animal model (Figure 7). The results demonstrated that topical application of KP-90 alleviated irradiation-induced dysregulation by promoting type I collagen synthesis and reducing collagen loss. KP-90 also suppressed the production and secretion of TNF-α, IL-1β, and IL-6, thereby mitigating inflammatory responses. Moreover, it downregulated the UV-induced overexpression of MMP-1, MMP-3, and MMP-9, limiting collagen fiber degradation. KP-90 further enhanced the activities of the antioxidant enzymes SOD, CAT, and GSH-Px, helping to neutralize skin ROS and alleviate UV-induced damage. In a related study, Kong et al. [23] conducted a similar experiment using chitosan oligosaccharide (COS). Topical application of COS on the dorsal skin of hairless mice after UV exposure for 10 weeks was found to increase the relative content of type I collagen, inhibit the levels of pro-inflammatory factors TNF-α, IL-1β, and IL-6, and significantly elevate the activities of the antioxidant enzymes SOD, GSH-Px, and CAT, thereby alleviating photoaging damage, whose effects potentially associated with the preservation of collagen morphology and content.

## 4. Materials and Methods

### 4.1. Materials and Reagents

*Kappaphycus alvarezii* was obtained from Wenchang, Hainan Province, China. Human immortalized keratinocytes (HaCaT cells) were obtained from Cell Resource Center, Shanghai Institute of Biological Sciences (Shanghai, China). Hyaluronic acid (sodium salt, Mw: 3–5 kDa, cosmetic grade) was purchased from Shanghai Macklin Biochemical Technology Co., Ltd. Dulbecco’s Modified Eagle’s Medium (DMEM), fetal bovine serum (FBS), trypsin, and other reagents for cell culture were purchased from Gibco Biotechnology Co., Ltd. (Grand Island, NY, USA). MTT kit, BCA assay kit and SOD kit was purchased from Nanjing Jiancheng Bio-Technology Co., Ltd. (Nanjing, China). Human ELISA kits of IL-1β, IL-6, TNF-α, MMP-1, MMP-3 and MMP-9 were obtained from Neobioscience Biotech, Co., Ltd. (Shenzhen, China). Female BALB/c nude mice (6–8 weeks old) were purchased from Zhuhai Baishitong Biotech Co., Ltd. (Zhuhai, China). Mouse ELISA kits of IL-1β, IL-6, TNF-α, MMP-1, MMP-3 and MMP-9 were obtained from mlbio Biotech Co., Ltd. (Shanghai, China). 100 Da molecular weight cut-off membrane was obtained from Shanghai Puke Biotechnology Co., Ltd. (Shanghai, China).

### 4.2. Preparation of Polysaccharide KP-90

*Kappaphycus alvarezii* was washed and air-dried until constant weight was achieved, then ground and passed through a 40-mesh sieve. The dried algal powder refluxed twice with 95% ethanol at a ratio of 1:4 (*w*/*v*) to remove pigments and other low-molecular-weight impurities. The resulting solid was air-dried at room temperature. The decolorized material was subsequently extracted with 0.135 mol/L citric acid solution at a solid-to-liquid ratio of 1:50 (*w*/*v*) at 100 °C for 4 h. After cooling to room temperature, the mixture was vacuum-filtered to remove insoluble residues. The filtrate was neutralized and concentrated to one-tenth of its original volume by rotary evaporation at 60 °C. The concentrate was dialyzed against deionized water using a 100 Da molecular weight cut-off membrane for 48 h, followed by lyophilization to yield the crude polysaccharide, designated as KP-0.

KP-0 was subsequently degraded using a UV/H_2_O_2_ free-radical system as previously described [39]. A solution of KP-0 (2.5 mg/mL) and H_2_O_2_ (400 mmol/L) in a glass dish was exposed to UV irradiation for 90 min to facilitate degradation, then eliminate residual H_2_O_2_. The solution was dialyzed again. The retained fraction was collected and lyophilized to obtain the degraded polysaccharide, labeled KP-90.

### 4.3. HaCaT Cell Experiments

#### 4.3.1. HaCaT Cell Culture

HaCaT cells were cultured according to the method described by Hu et al. [15]. The cells were seeded in culture flasks and maintained in DMEM complete medium supplemented with 10% fetal bovine serum and 1% penicillin–streptomycin. Cells were passaged at approximately 30% confluence, with a routine subculturing interval of two days. All cultures were incubated at 37 °C in a humidified atmosphere containing 5% CO_2_. When cells reached appropriate density, they were detached using trypsin. Specifically, the old medium was aspirated, and the cell layer was rinsed with PBS buffer, which was then removed. Trypsin was added until cell rounding and detachment were observed, at which point complete medium was introduced to stop the reaction. Gentle mechanical agitation and pipetting were applied to ensure complete cell detachment. The cell suspension was transferred to a centrifuge tube for counting. A portion of the harvested cells was used for further passaging, and the remainder was allocated for subsequent experiments.

#### 4.3.2. HaCaT Cell Viability Assessed by MTT Assay

Cytotoxicity was evaluated using the MTT assay as previously reported [15]. In brief, HaCaT cells were seeded into 96-well plates at a density of 1 × 10^4^ cells per well. After 24 h of incubation, the medium was replaced with serum-free DMEM for 12 h of starvation. Cells were then treated with different solutions: the control and model groups received basal medium, while experimental groups were treated with various concentrations of KP-90 dissolved in basal medium. Following 12 h of treatment, cell viability was determined by measuring the absorbance at OD_570_ after MTT incubation, and the results were expressed as a percentage of viable cells.

For the UVB irradiation experiment, cell viability was assessed similarly. After 12 h of starvation, the medium was replaced with PBS. All groups except the control were exposed to UVB radiation at a dose of 3 mJ/cm^2^. After irradiation, PBS was replaced with complete medium, and cells were cultured for another 24 h. The MTT assay was then performed as described above.

#### 4.3.3. Collection of HaCaT Cell Supernatant and Cell Lysis

Based on the modeling approach outlined in Section 4.3.2, with minor modifications, HaCaT cells were seeded in 6-well plates at 5 × 10^5^ cells per well. Experimental groups were treated with KP-90 at concentrations of 125, 250, and 500 μg/mL, while 500 μg/mL hyaluronic acid was used as a positive control. After 24 h of treatment, the culture supernatant was collected. Cells were then lysed by adding a quantified amount of lysis buffer and incubating on ice for 20 min. Residual cells were scraped off using a cell scraper, transferred to EP tubes, and further lysed on ice for another 20 min. After centrifugation, the supernatant was collected as the cell lysate. Protein concentration in the lysate was determined using a BCA protein assay kit which was purchased from Nanjing Jiancheng Bio-Technology Co., Ltd. (Nanjing, China).

#### 4.3.4. Measurement of Cellular Indicators in HaCaT

The collected cell supernatant from Section 4.3.3 was used to determine the levels of matrix metalloproteinases (MMP-1, MMP-3, and MMP-9) and pro-inflammatory cytokines (TNF-α and IL-6) according to the manufacturer’s instructions. Additionally, the activity of the antioxidant enzyme SOD was measured using the cell lysate described in Section 4.3.3.

### 4.4. HDF Cell Experiments

#### 4.4.1. HDF Cell Culture

Refer to Section 4.3.1 for the general procedure, with the following modifications: HDF cells were cultured in DMEM complete medium containing 15% fetal bovine serum and 1% penicillin–streptomycin. Cells were passaged at a density no less than 50%, with a standard culture period of 7 days.

#### 4.4.2. HDF Cell Viability Assessed by MTT Assay

Cytotoxicity was evaluated using the MTT assay as described in Section 4.3.2. Particularly, HDF cells were seeded in 96-well plates at a density of 8 × 10^3^ cells per well. All groups except the control were exposed to UVA radiation at a dose of 9 J/cm^2^.

#### 4.4.3. Collection of HDF Cell Supernatant and Cell Lysis

Referring to the cell modeling method in Section 4.4.2 with minor adjustments, HDF cells were seeded in 6-well plates at 6 × 10^5^ cells per well and treated with KP-90 (62.5, 125, and 250 μg/mL) and hyaluronic acid (250 μg/mL) for 72 h. The cell culture supernatant and cell lysate were collected following the procedure described in Section 4.3.3.

#### 4.4.4. Measurement of Cellular Indicators in HDF

The collected cell supernatant from Section 4.4.3 was used to determine the levels of procollagen type I α1 chain, hyaluronic acid, and malondialdehyde according to the manufacturer’s instructions. Additionally, the activities of the antioxidant enzymes SOD, CAT, and GSH-Px were measured using the cell lysate obtained in Section 4.4.3.

### 4.5. Franz Diffusion Cell

An in vitro Franz diffusion cell model was established using neonatal porcine skin, with an effective diffusion area of 2.32 cm^2^. The donor solution contained the test sample at a concentration of 10 mg/mL, while the receptor compartment was filled with purified water maintained at 32 ± 1 °C. At the start (0 h), 1 mL of the sample solution was applied to the donor chamber. Subsequently, 0.5 mL of the receptor fluid was collected at 1, 2, 3, 4, 6, 8, 10, and 24 h, and immediately replaced with an equal volume of fresh distilled water. After 24 h, the experiment was terminated. The total sugar content in each collected sample was determined using the phenol–sulfuric acid method, and the skin permeation was calculated based on the following formula:

(1) $$Absorption=\frac{Total\;mass\;of\;permeated\;sugars/\mu g}{Skin\;diffusion\;area/{\mathrm{cm}}^{2}}$$

### 4.6. Animal Housing

All animal procedures were approved by the Animal Ethics Committee of South China University of Technology (Approval No. AE-2025018). The animals were housed in the university’s Laboratory Animal Center, which holds the Animal Use License No. SYXK (Yue) 2022-0178. The animal license number provided was SCXK (Yue) 2018-0002.

**Figure 8:**
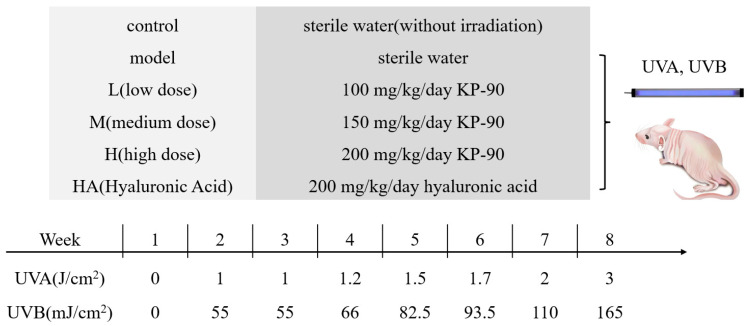
Schematic diagram of experimental procedure.

Housing conditions were maintained under a standardized specific pathogen-free (SPF) environment, with a room temperature of 22 ± 2 °C, relative humidity of 55–70%, and a 12 h light/12 h dark cycle. After acclimatization period, 60 BALB/c nude mice were randomly divided into 6 groups (*n* = 10 per group): control, model, HA, L, M, and H. The specific treatment conditions for each group are summarized in Figure 8.

KP-90 and hyaluronic acid were dissolved in sterile deionized water to prepare stock solutions at concentrations of 10, 15, and 20 mg/mL (for the L, M, and H groups, respectively) and 20 mg/mL (for the HA group). All solutions were filtered through a 0.22 μm sterile membrane prior to application. A fixed volume of 200 μL of the respective solution was evenly applied to the dorsal skin using a sterile pipette and gently spread with the side of the tip to ensure uniform coverage. Applications were performed daily, 1 h before UV irradiation. The control and model groups received daily topical applications of sterile water at a volume of 200 μL per mouse.

### 4.7. Establishment of Photoaging Mice Model

With the exception of the control group, all mice were irradiated three times per week (Monday, Wednesday, Friday) for 7 consecutive weeks using a UVA/UVB photoaging device (UVA: 1660 μW/cm^2^; UVB: 91.7 μW/cm^2^) equipped with two UVA lamps (365 nm) and one UVB lamp (313 nm), positioned 20 cm above the dorsal skin. The UVA dose per session was 34.2 J/cm^2^, and the UVB dose per session was 1.88 J/cm^2^. The total cumulative doses over the 7-week period were 718.2 J/cm^2^ for UVA and 39.48 J/cm^2^ for UVB.

### 4.8. Measurement of TEWL

On the day prior to the conclusion of the experiment, *TEWL* values of the dorsal skin were measured using a VapoMeter^®^. For each mouse, three distinct sites (upper, middle, and lower) on the dorsal skin were selected, and three repeated measurements were taken at each site. The mean value was calculated for analysis.

### 4.9. Hematoxylin and Eosin (H&E) Staining

Skin sections were stained with H&E, observed under an optical microscope, and imaged. The epidermal and dermal thicknesses were semi-quantitatively analyzed and measured using ImageJ 1.x software (National Institutes of Health, Bethesda, MD, USA). Epidermal thickness was measured at 10 randomly selected interfollicular sites per section. Dermal thickness was measured from the dermo-epidermal junction to the subcutaneous fat layer.

### 4.10. Masson Staining

Skin sections were stained with Masson’s trichrome, imaged under a light microscope, and analyzed using ImageJ 1.x software. The collagen fibers were identified and quantified to calculate the CVF, defined as the percentage of the total tissue area-stained blue for collagen. CVF was calculated as the percentage of blue-stained area relative to the total dermal area using color deconvolution plugin.

### 4.11. Preparation of Skin Homogenates and Measurement of Related Indicators

A precisely weighed portion of skin tissue was placed into a nuclease-free grinding tube, and its weight was recorded using an analytical balance. A pre-cooled PBS solution was added at a 1:9 (*w*/*v*) ratio, followed by the addition of three 3 mm zirconium oxide grinding beads. The mixture was homogenized using a cryogenic tissue grinder under the following conditions: frequency of 70 Hz, temperature maintained at −10 °C, with 20 cycles of 60 s grinding followed by 20 s pauses. After homogenization, the sample was centrifuged at 12,000× *g* for 10 min to collect the supernatant. The supernatant was aliquoted to avoid repeated freeze–thaw cycles and stored at −40 °C for subsequent analysis. The levels of type I collagen, MMP-1, MMP-3, MMP-9, TNF-α, IL-6, and IL-1β, as well as the enzymatic activities of SOD, CAT, and GSH-Px, were determined according to the instructions provided with the respective assay kits.

### 4.12. Statistical Analysis

Data are presented as mean ± standard deviation (SD) from at least three independent replicates. Statistical significance was determined by one-way analysis of variance (ANOVA) followed by Tukey’s post hoc test using SPSS 27 software (IBM, New York, NY, USA). A *p*-value of less than 0.05 was considered statistically significant. Graphs were generated using GraphPad Prism 9 (GraphPad Software, La Jolla, CA, USA).

## 5. Conclusions

In this study, a low-molecular-weight polysaccharide, KP-90, was extracted and prepared from *Kappaphycus alvarezii*. Its anti-photoaging activity was first evaluated in vitro using UVB-induced HaCaT cells and UVA-induced HDF cells, and further validated in a UVA/UVB-induced nude mice model. The results demonstrated that KP-90 contributes to reducing irradiation-induced epidermal thickening, enhancing the synthesis of type I collagen and hyaluronic acid, and alleviating skin laxity, dryness, roughness, and wrinkle formation. These beneficial effects were achieved through multiple mechanisms, including enhanced cell viability, reduced generation of ROS and MDA, restoration of antioxidant enzyme activities (SOD, CAT, GSH-Px), downregulation of matrix metalloproteinases (MMP-1, MMP-3, MMP-9), and suppression of the synthesis and secretion of inflammatory cytokines (TNF-α, IL-6, IL-1β). These findings provide a theoretical foundation for developing KP-90 as a functional ingredient in anti-photoaging skincare products, and also support the comprehensive utilization of marine bio-resources, thereby contributing to the growth of the blue bioeconomy.

## Data Availability

The raw data supporting the conclusions of this article will be made available by the authors on request.
